# Prefrontal Cortex Oxygenation During Endurance Performance: A Systematic Review of Functional Near-Infrared Spectroscopy Studies

**DOI:** 10.3389/fphys.2021.761232

**Published:** 2021-10-26

**Authors:** Jonas De Wachter, Matthias Proost, Jelle Habay, Matthias Verstraelen, Jesús Díaz-García, Philip Hurst, Romain Meeusen, Jeroen Van Cutsem, Bart Roelands

**Affiliations:** ^1^Human Physiology and Sports Physiotherapy Research Group, Vrije Universiteit Brussel, Brussels, Belgium; ^2^Faculty of Sport Sciences, University of Extremadura, Caceres, Spain; ^3^The School of Psychology & Life Sciences, Canterbury Christ Church University, Canterbury, United Kingdom; ^4^VIPER Research Unit, Royal Military Academy, Brussels, Belgium

**Keywords:** near-infrared spectroscopy (NIRS), endurance exercise, prefrontal cortex, oxygenation, respiratory compensation point (RCP), systematic review

## Abstract

**Introduction:** A myriad of factors underlie pacing-/exhaustion-decisions that are made during whole-body endurance performance. The prefrontal cortex (PFC) is a brain region that is crucial for decision-making, planning, and attention. PFC oxygenation seems to be a mediating factor of performance decisions during endurance performance. Nowadays, there is no general overview summarizing the current knowledge on how PFC oxygenation evolves during whole-body endurance performance and whether this is a determining factor.

**Methods:** Three electronic databases were searched for studies related to the assessment of PFC oxygenation, through near-IR spectroscopy (NIRS), during endurance exercise. To express PFC oxygenation, oxygenated (HbO_2_) and deoxygenated hemoglobin (HHb) concentrations were the primary outcome measures.

**Results:** Twenty-eight articles were included. Ten articles focused on assessing prefrontal oxygenation through a maximal incremental test (MIT) and 18 focused on using endurance tasks at workloads ranging from low intensity to supramaximal intensity. In four MIT studies measuring HbO_2_, an increase of HbO_2_ was noticed at the respiratory compensation point (RCP), after which it decreased. HbO_2_ reached a steady state in the four studies and increased in one study until exhaustion. All studies found a decrease or steady state in HHb from the start until RCP and an increase to exhaustion. In regard to (non-incremental) endurance tasks, a general increase in PFC oxygenation was found while achieving a steady state at vigorous intensities. PCF deoxygenation was evident for near-to-maximal intensities at which an increase in oxygenation and the maintenance of a steady state could not be retained.

**Discussion/Conclusion**: MIT studies show the presence of a cerebral oxygenation threshold (ThCox) at RCP. PFC oxygenation increases until the RCP threshold, thereafter, a steady state is reached and HbO_2_ declines. This study shows that the results obtained from MIT are transferable to non-incremental endurance exercise. HbO_2_ increases during low-intensity and moderate-intensity until vigorous-intensity exercise, and it reaches a steady state in vigorous-intensity exercise. Furthermore, ThCox can be found between vigorous and near-maximal intensities. During endurance exercise at near-maximal intensities, PFC oxygenation increases until the value exceeding this threshold, resulting in a decrease in PFC oxygenation. Future research should aim at maintaining and improving PFC oxygenation to help in improving endurance performance and to examine whether PFC oxygenation has a role in other performance-limiting factors.

## Introduction

Dynamic endurance exercise can be defined as prolonged (>75 s) exercise and can be classified into whole-body endurance and local muscle endurance (Mccormick et al., 2015). Dynamic whole-body endurance exercise involves large muscle groups (e.g., cycling, running, and rowing), whereas muscle endurance exercise involves only one muscle or muscle group (e.g., knee extension or handgrip tasks) (Kenney et al., 2012; Pageaux et al., 2013; Mccormick et al., 2015). In general, whole-body endurance tasks are most frequently measured during time-to-exhaustion (TTE), time-trials (TTs) (Amann et al., 2008), and constant intensity fixed duration (CIFD) tasks (i.e., both intensity and duration are fixed factors) (Ichinose et al., 2020). In contrast to TT and TTE performance, CIFD tasks are mainly used to assess psychological (e.g., perceived exertion rating, thermal discomfort, etc.) and physiological (e.g., heart rate (HR), blood lactate, etc.) reactions during exercise.

During both TTE and TTs, important decisions that impact performance need to be taken into consideration. In TTE, participants need to decide when to stop (e.g., “*Am I totally exhausted and have to stop this exercise?”*) and in TTs, participants need to decide how much effort to give (e.g., “*When should I slow down/speed up to reach the set goal as fast/good as possible?”*). A large body of research has examined the underlying mechanisms of these decisions. Périard and Racinais (2015) suggested that decisions during TTs in hypoxic- and hot conditions are related to oxygen availability, whereas Girard and Racinais (2014) reported that decisions during TTE are related to alterations in central command. Similarly, core temperatures exceeding > 39°C and oxygen saturation dropping to less than < 70% O_2_ saturation have been associated with performance-related decisions in both TT and TTE (Racinais and Girard, 2012). Psychobiological factors are also likely to play a role. Van Cutsem et al. (2019) reported an increase in subjective thermal strain resulted in a decrease in performance (i.e., an earlier exhaustion-decision). Given this evidence, it is clear that a myriad of factors plays a role in the underlying mechanisms of decisions in both TTE and TTs.

All the proposed mediating factors that play a role in decisions in TTE and TTs are likely to be located both peripherally (i.e., muscles) and centrally (i.e., the brain and central nervous system). Peripherally, for example, locomotor muscle fatigue is mediated by the accumulation of intracellular metabolites, which eventually cause failure in excitation-contraction coupling (Amann, 2011). Whereas centrally, it is theoretically hypothesized that the corollary discharge model determines the perception of effort by sending a copy of a motor command to the somatosensory areas. These corollary discharges influence performance decisions about how much effort is needed to give and highlight the brain as an important mediating factor in decision-making in both TTs and TTE.

The top-downregulation of the prefrontal cortex (PFC) during exercise tolerance and volition is also likely to play a role in performance decisions (Robertson and Marino, 2016). After applying 30-min transcranial direct current stimulation to the dorsolateral PFC, Angius et al. (2019) reported improvements in TTE, lower ratings of perceived exertion (RPE) and HR, and higher blood lactate than no-treatment controls. Angius et al. (2019) concluded that by targeting PFC using transcranial direct current stimulation, improvements in performance were the result of a change in decision-making, planning, attention, (short-term) memory, and executive function. A body of research has shown that PFC is an underlying mechanism for the termination of whole-body endurance exercise and is mediated by the oxygenation of the cerebral cortex (Ide et al., 1999; Ide and Secher, 2000; Bhambhani et al., 2007; Rupp and Perrey, 2008; Rooks et al., 2010). It is likely that PFC oxygenation during incremental exercise increases from low-to-hard intensities, and declines to preceding exhaustion (Rooks et al., 2010).

As mentioned earlier, PFC oxygenation seems to be an important and promising mediating factor of performance decisions during whole-body endurance performance. The two techniques that can be used to assess PFC oxygenation are functional MRI (fMRI) and functional near-IR spectroscopy (fNIRS). fMRI is suggested to be the gold standard for measuring changes in brain oxygenation and provides a high spatial resolution (up to 4.0 mm) that, in turn, can help to measure functional hemodynamic changes in the brain (Glover, 2011; Herold et al., 2018). On the other hand, fMRI is expensive and results in relatively low temporal resolution (≈0.5 Hz) due to its sensitivity to the movement (Ekkekakis, 2009; Herold et al., 2018). fNIRS is a non-invasive optical imaging technique that measures relative changes in hemoglobin (Hb) concentrations. Compared to fMRI, fNIRS provides researchers with more possibilities to examine performance in ecological settings given that it is less sensitive to movement. However, the spatial resolution for fNIRS (≈10–20 mm) is inferior to that for fMRI (Glover, 2011; Scarapicchia et al., 2017). This disadvantage has, however, not held back researchers from attempting to create further insights into the role of (prefrontal) brain oxygenation in performance-related decisions during whole-body endurance performance (e.g., Rooks et al., 2010; Leff et al., 2011). Rooks et al. (2010) reported that PFC oxygenation during incremental exercise increased between moderate and hard intensities after which it drastically decreased at very hard intensities. Leff et al. (2011) replicated these results and reported that cortical oxygenation increased during the first few minutes following exercise until near-maximal exercise and a decrease in PFC oxygenation at near-maximal to intense exhaustive exercise. Herold et al. (2018) summarized the methodological knowledge of fNIRS, outlined recommendations for future research (Herold et al., 2018), and confirmed the relevance and applicability of fNIRS during exercise science. However, it has been over a decade since Rooks et al. (2010) summarized the effects of incremental exercise on cerebral oxygenation. To date, there is no general overview summarizing current knowledge on how PFC oxygenation evolves during (non-incremental) whole-body endurance exercise at different intensities and whether this influences performance. Therefore, there is a need for a state-of-the-art summary of cerebral oxygenation during whole-body endurance performance.

As mentioned earlier, it is clear that fNIRS research can help elucidate the underlying mechanisms of performance-related decisions during endurance exercise. To advance the field of whole-body endurance performance and highlight the opportunities to evaluate the impact of specific countermeasures on the performance-related changes in fNIRS variables, the aim of this systematic review is to provide an updated review of the extant research. We hypothesize that: (1) prefrontal oxygenation increases during submaximal exercise and subsequently decreases at a near-maximal intensity, (2) prefrontal oxygenation increases during prolonged submaximal exercise until a steady state is reached, and (3) at near-maximal intensities PFC oxygenation cannot be maintained, resulting in a decrease near exhaustion.

## Methods

This systematic review followed the guidelines provided by the Preferred Reporting Items for Systematic Review and Meta-Analyses statement (Page et al., 2021).

### Eligibility Criteria

To determine keywords for a review, we used the participants, intervention, comparison, outcome, and study design (PICOS) search tool (see Table 1). Studies with a healthy human adult population, aged between 18 and 45 years old, and without gender restrictions were included. Given that an aerobic energy system predominates during maximum effort exercise after 75 s (Gastin, 2001), we included the studies using dynamic whole-body endurance performance with a duration of at least 75 s. Maximal incremental exercise tasks were also included in this review. The two main outcome parameters for inclusion were performance on the exercise task and PFC oxygenation. A baseline measure at the start of the exercise-task had to be made for cerebral oxygenation to be able to evaluate the evolution of oxygenation throughout the trials. Randomized controlled trials (RCTs), non-RCTs (nRCTs), or non-randomized non-controlled trials (nRnCTs), written in English, were considered to be eligible for inclusion. Studies were excluded when the study population was patients who were using medications. Additionally, animal studies, individual case studies, and interventions where participants performed with their eyes closed were also excluded.

**TABLE 1:** “PICOS” categories (participants, interventions, comparisons, outcomes, study design) used to determine keywords.

PICOS component	Detail
Participants	Healthy humans (18–45 years)
Interventions	Whole-body endurance performance task (>75 s)
Comparisons	Baseline NIRS-measures before the start of and/or during the whole-body endurance performance task or the control condition (e.g., exercise modality)
Outcomes	Prefrontal cortex oxygenation (NIRS) during whole-body endurance task or performance
Study designs	RCTs, nRCT, nRnCTs

*NIRS, Near infrared spectroscopy RCT randomized controlled trial; nRCT non-randomized controlled trials; nRnCTs non-randomized non-controlled trials.*

### Information Sources and Search Strategy

Three electronic databases, PubMed, Web of Science, and Cochrane Library (until August 20, 2021), were searched with no date restrictions. Medical subject heading (mesh) terms, if available in PubMed and Cochrane Library, were used for a qualitative literature search. The following keywords were applied individually or in combination: Near Infrared Spectroscopy, NIRS, Magnetic Resonance Imaging, MRI, Near Infrared Spectroscopy [MeSH], Magnetic Resonance Imaging [MeSH], Physical performance, Exercise performance, Endurance performance, Time trial, Time to exhaustion, physical endurance, Athletic performance, physical endurance [MeSH], Athletic performance [MeSH], Motor Activity [MeSH], Oxygenation, BOLD response, hemodynamic response, Cerebrovascular Circulation, Cerebrovascular Circulation [MeSH] prefrontal cortex OR frontal lobe OR frontal lobe [MeSH] (Table 2). The combination of keywords 1, 2, and 3 (see Table 2) was included in a search strategy. In addition, to ensure the literature saturation, a backward search and a forward search were performed by screening the reference lists of the included articles and by screening the citations of the included studies, respectively, to increase the likelihood of the inclusion of all relevant studies.

**TABLE 2:** Search strategy: number of hits on keywords and combined keywords in PubMed, Web of Science, and Cochrane Library.

Keywords	PubMed	Web of science	Cochrane library
	No. of hits (20/08/2020)	No. of hits (20/08/2020)	No. of hits (20/08/2020)
(1) Near infrared spectroscopy OR NIRS OR magnetic resonance imaging OR MRI or near infrared spectroscopy [MeSH] OR magnetic resonance imaging [MeSH]	608,218	487,775	33,168
(2) Physical performance OR exercise performance OR endurance performance OR time trial OR time to exhaustion OR physical endurance OR athletic performance OR physical endurance [MeSH] OR athletic performance [MeSH] OR motor activity [MeSH]	336,117	137,120	31,398
(3) Oxygenation OR BOLD response OR hemodynamic response OR cerebrovascular circulation OR cerebrovascular circulation [MeSH]	124,655	77,973	51,012
(4) prefrontal cortex OR frontal lobe OR frontal lobe [MeSH]	107,239	118,480	7524
**Combined key words**			
(1) AND (2)	6566	549	1664
(1) AND (2) AND (3)	4650	389	352
(1) AND (2) AND (3) AND (4)*	921	202	335
(1) AND (2) AND (4)	1408	48	350

Combined keywords with a “*” were included in the study.

### Study Selection and Data Collection Process

**FIGURE 1:**
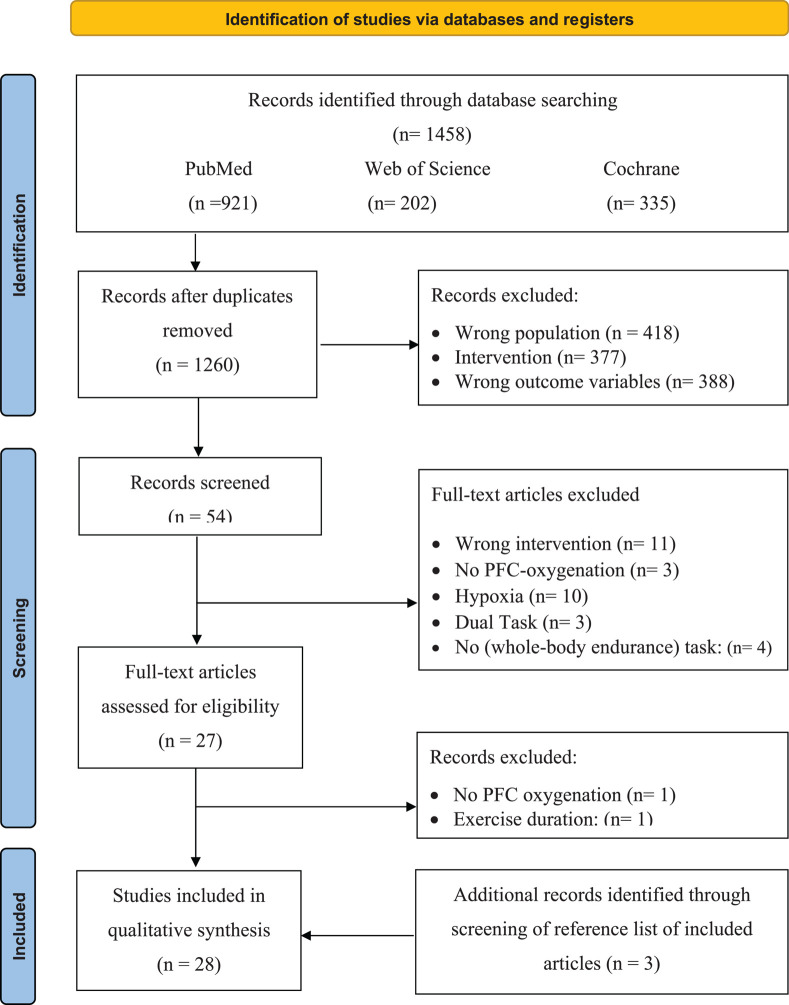
Selection process for research articles (*n* = 28) included in this systematic review. Adapted version of the recommendations in the PRISMA 2020 statement (Liberati et al., 2009). *nRCT*, non-randomized controlled trial; *nRnCT*, non-randomized non-controlled trial; *RCT*, randomized controlled trial.

Articles from the three databases were collated in EndNote X9 where the duplicates were removed (Table 3). Afterward, all studies were imported into Rayyan (the web and mobile app for systematic reviewers; Ouzzani et al., 2016), where the two reviewers (JDW and JH), independently and blinded from each other, screened the title and abstracts for each study. The search resulted in 89 (7%) conflicting studies. After the conflicts were resolved, the full text of the remaining articles was screened. A general meeting with the research team was held to decide on inclusion. The full-text version of all the articles that met the inclusion criteria (Table 3) was retrieved for quality assessment and data extraction (see quality assessment). If, after this screening, a citation was considered potentially eligible for inclusion and relevant, the full-text article was retrieved. A flow diagram illustrating the selection of the included studies can be found in Figure 1.

**TABLE 3:** Inclusion and exclusion criteria.

Inclusion	Exclusion
fNIRS	Individual case studies
Healthy participants (18–45 years)	Animal studies
Neutral ambient conditions	Eyes closed
Cycling/running/rowing	Psychiatric disorders/patients
Whole-body endurance performance task	Medication

*fNIRS, functional Near-Infrared Spectroscopy.*

### Quality Assessment

Methodological quality was assessed using the quantitative assessment tool “QualSyst” (Kmet and Lee, 2004). The QualSyst tool is a checklist containing 14 items, which are plotted on a 3-point scale (yes = 2, partial = 1, and no = 0). Items that were not applicable to a particular study design were marked as “n/a” and were excluded from the calculation of a summary score (Kmet and Lee, 2004). A quality score was calculated for each paper by summing the total score obtained across relevant items and dividing them by the total possible score (Kmet and Lee, 2004). The two reviewers (JDW and JH) independently performed quality assessments, and disagreements were solved through a consensus. A score of ≥ 75%, 55–75%, and ≤ 55% indicated strong quality, moderate quality, and weak quality, respectively.

### Classification of Intensity

Based on the ACSM position stand, the classification of exercise intensity: relative and absolute exercise intensity for cardiorespiratory endurance and resistance exercise, whole-body endurance performance tasks were classified into very light, light, moderate, vigorous, and near-maximal intensities (Garber et al., 2011). The classification, where necessary, was based on energy cost calculations using the ACSM energy equation for cycling (Swain, 2000).

## Results

### Study Selection

After the removal of duplicates, the search strategy resulted in 1,260 articles. After screening the titles and abstracts of the remaining 54 (Figure 1), 25 met the inclusion criteria. After screening the reference lists of the full-text articles additional three articles were included, resulting in a total of 28 articles, of which 53% were scored as strong, 36% as moderate, and only 11% as weak on the quality assessment (as shown in Table 4).

**TABLE 4:** Quality assessment “QualSyst” (Kmet and Lee, 2004).

Study	A	B	C	D	E	F	G	H	I	J	K	L	M	N	Rating*
Shibuya et al. (2004)	2	2	1	2	NA	NA	NA	2	1	2	2	NA	2	2	Strong: 90
Suzuki et al. (2004)	1	1	1	2	NA	NA	NA	1	1	2	1	NA	2	1	Moderate: 65
Billaut et al. (2010)	2	2	2	2	NA	NA	NA	1	1	2	2	NA	2	2	Strong: 90
Fumoto et al. (2010)	1	1	2	2	NA	NA	NA	1	1	1	2	NA	1	2	Moderate: 70
Keramidas et al. (2011)	2	1	1	2	0	0	NA	1	1	2	2	1	1	2	Moderate: 62
Miyazawa et al. (2013)	2	1	1	2	NA	NA	NA	1	1	2	2	NA	1	2	Strong: 75
Rupp et al. (2013)	2	1	1	2	NA	NA	NA	2	1	2	2	NA	1	1	Strong: 75
Giles et al. (2014)	2	1	2	2	0	0	0	2	1	1	1	1	2	2	Moderate: 61
Santos-Concejero et al. (2015)	2	2	2	2	NA	NA	NA	2	1	2	1	NA	1	2	Strong: 85
Pires et al. (2016)	2	2	2	2	NA	NA	NA	2	1	2	2	NA	2	2	Strong: 95
Santos-Concejero et al. (2017)	2	1	2	2	NA	NA	NA	2	1	2	2	NA	2	2	Strong: 90
Ohyanagi et al. (2018)	2	0	0	0	0	NA	NA	1	1	1	2	1	1	2	Weak: 46
Radel et al. (2017)	2	2	2	2	0	1	2	2	2	2	0	1	2	2	Strong: 79
Tsubaki et al. (2018)	2	2	1	1	NA	NA	NA	1	1	2	1	NA	2	1	Moderate: 70
Kounalakis and Geladas (2012)	2	2	1	2	2	0	0	2	1	1	2	2	2	2	Strong: 75
Takehara et al. (2017)	2	0	1	2	0	0	0	1	1	1	0	1	2	2	Weak: 46
Ide et al. (1999)	1	0	1	2	NA	NA	NA	2	1	1	2	NA	2	1	Moderate: 65
Bhambhani et al. (2007)	2	2	1	2	NA	NA	NA	2	1	1	2	NA	2	2	Strong: 85
Rupp and Perrey (2008)	2	2	1	2	NA	NA	NA	2	1	2	1	NA	2	2	Strong: 85
Timinkul et al. (2008)	2	2	1	2	NA	NA	NA	2	1	2	2	NA	2	2	Strong: 90
Tempest et al. (2014)	2	2	2	2	NA	NA	NA	2	2	2	2	NA	2	2	Strong: 100
Jung et al. (2015)	2	2	1	0	NA	NA	NA	2	1	1	2	NA	1	1	Moderate: 65
Oussaidene et al. (2015)	2	2	2	2	NA	NA	NA	2	1	2	2	NA	2	2	Strong: 95
Tempest and Parfitt (2016)	2	2	2	2	NA	NA	NA	2	2	1	2	NA	2	2	Strong: 95
Tempest et al. (2017)	2	2	1	1	0	0	NA	2	1	1	2	1	2	2	Moderate: 65
Stevens et al. (2018)	1	1	1	1	2	0	1	2	1	2	2	2	2	2	Moderate: 71
Hiura et al. (2018)	2	0	1	1	NA	NA	NA	2	1	1	2	NA	2	2	Moderate: 70
Kojima et al. (2020)	2	1	1	1	NA	NA	NA	1	0	1	0	NA	0	1	Weak: 40
Kojima et al. (2021)	2	1	1	1	NA	NA	NA	1	1	1	1	NA	2	1	Moderate: 60

A, Objective sufficiently described; B, evident study design?; C, Method of subject/comparison group selection or source of information/input variables described and appropriate?; D, Subject (and comparison group, if applicable) characteristics sufficiently described?; E, If interventional and random allocation was possible, was it described?; F, If interventional and blinding of investigators was possible, was it reported?; G, If interventional and blinding of subjects was possible, was it reported?; H, Outcome and (if applicable) exposure measure(s) well de ned and robust to measurement / misclassification bias? means of assessment reported?; I, Sample size appropriate?; J, Analytic methods described/justified and appropriate?; K, Some estimate of variance is reported for the main results?; L, Controlled for confounding?; M, Results reported in sufficient detail?; N, Results support conclusion?. *Quality scores: ≥ 75% strong, 55 ≥ 75% moderate, ≤ 55% weak.

### Characteristics of Near-IR Spectroscopy

Positioning of the optodes and receivers with the associated inter-optode distances (IODs) varies and is described in Tables 5A,B. Nineteen studies used the international EEG 10-20 system with optode placements over the prefrontal lobe at Fp1,Fp2, Fp3, and/or Fp4 position (Rupp and Perrey, 2008; Timinkul et al., 2008; Billaut et al., 2010; Keramidas et al., 2011; Rupp et al., 2013; Giles et al., 2014; Oussaidene et al., 2015; Santos-Concejero et al., 2015, 2017; Pires et al., 2016; Tempest and Parfitt, 2016; Tsubaki et al., 2016, 2018, 2020; Takehara et al., 2017; Tempest et al., 2017; Asahara and Matsukawa, 2018; Ohyanagi et al., 2018; Stevens et al., 2018) pecifying this location as ± 3 cm from the midline, just above the supra-orbital ridge (Bhambhani et al., 2007; Miyazawa et al., 2013). Jung et al. (2015) used Broadman area 10, Montreal Neurophysiological Institute (MNI) coordinates [(*x*/*y*/*z*) −40, 50, 0], two studies performed a three-dimensional T1-weighted MRI scan, marking the optode location for the left and right PFC (Suzuki et al., 2004; Fumoto et al., 2010), and finally, two studies placed the device on the participants’ forehead without specifying the exact location of the optode placement (Shibuya et al., 2004; Kounalakis and Geladas, 2012). The IODs varied between 25, 30, 38, 40, 45, and 50 mm (mean ± SD: 38 ± 7 mm).

**TABLE 5A:** Overview of the results within “non-incremental endurance exercise”.

Study	Sample characteristics (Mean ± SD)	NIRS—devise, Inter Optode Distance (IOD), position, wave specifications	Physical task	Type of task and exercise intensity	Outcome
Shibuya et al. (2004)	5 M A: 24.6 ± 0.4 year Length: 175.3 ± 1.2 cm Mass: 62.9 ± 1.1 kg VO_2peak_: 48.4 ± 1.3 ml/kg/min Training status: /	IOD: 50 mm Position: forehead 780, 810, and 830 nm	(Cycling) 120% VO_2peak_ exhaustive exercise test	TTE – 120% VO2peak – *Supramaximal*	• SaO_2_ = throughout test • VO_2_, V_E_, and HR ↑ gradually over time • [HbO_2_] ↑ first 30 s • [tHb] and [HbO_2_] = throughout test • End of exercise: [tHb] and [HbO_2_] ↓ from pre-exercise level
Suzuki et al. (2004)	7 M, 2 F A: 28.1 ± 7.4 years Training status: /	OMM-2001, Shimadzu, Kyoto, Japan IOD: / Position: anatomical 3-D T1 weighted MRI scan was performed, marking the optode location 780, 805, and 830 nm Continuous wave	(Running) 3 treadmill locomotor tasks of 90 s. each following 30 s rest: 1. Walking at 3 km/h 2. Walking at 5 km/h 3. Running at 9 km/h Randomized order	Adapting speed – 3 km/h 5 km/h 9 km/h – *Light to moderate*	• HbO_2_ and tHb ↑ bilaterally before starting the locomotor tasks especially at 9 km/h and peaked before the treadmill speed got steady. • After reaching constant speed: HbO_2_ and tHb ↓ and tended to return to the baseline or below baseline during performing the locomotor tasks. • After stopping locomotion: temporal drops in HbO_2_ before returning to the baseline •↑ in HbO_2_ levels greatest at 9 km/h but are = between 3 and 5 km/h. • PFC-activation = greater during running at 9 km/h vs. walking at 3 and 5 km/h
Billaut et al. (2010)	11 M A: 24.8 ± 4.2 Length: 174.2 ± 5.3 cm Mass: 66.8 ± 3.1 kg Fat%: 10.3 ± 3.5% Training status: PL5	Oxymon MKIII; Artinis Medical Systems b.v., Zetten, the Netherlands IOD: 45 mm Position: Fp1 and F3, according to the modified international EEG 10–20 system 763 and 855 nm Continuous wave	(Running) T1: Familiarization trial T2: 5 km TT • 6 min self-paced warm up • 5 km TT (4°incline)	TT – Self-paced – *Near to maximal*	• HR: ↑ rapidly but remained nearly constant between 1.5 and 4 km and peaked in the last 0.5 km • SaO2: fell between 3 and 5 km • RPE ↑ throughout the trial • RBV and Cox ↑ until 2.5 km (↑△[HbO_2_], ↑△[HHb], and ↑△[tHb]), o = between 2.5 and 4.5 km, f o Deoxygenation in the last 0.5 km, while RBV remained stable
Fumoto et al. (2010)	9 M, 1 F A: 32 ± 2.2 Length: 174.2 ± 5.3 cm Mass :66.8 ± 3.1 kg Fat%: 10.3 ± 3.5 Training status: PL2	OMM3000; Shimadzu Co., Kyoto, Japan IOD: 30 mm Position: anatomical 3-D T1weighted MRI scan was performed, marking the optode location 780, 805, and 830 nm	(Cycling) 15 min at 60 RPM and pre-determined intensity of 12–13 RPE	Constant load with fixed duration – 12-13 RPE – *Moderate*	• Gradual ↑ in HbO_2_ and tHb, reached a steady state at the end of PE (mainly in ventral PFC regions) • Gradual ↓ in HbO_2_ and tHb were observed after stopping • Small ↓ in HHb or no change in both the ventral and dorsal PFC
Miyazawa et al. (2013)	10 M A: 20.0 ± 1 year Length: 170 ± 5 cm Mass: 64 ± 9 kg Training status: /	NIRO200, Hamamatsu Photonics, Hamamatsu, Japan IOD : 40 mm Position: left forehead, ± 3 cm from midline, just above supra-orbital ridge 780 nm	(Cycling) • 4 min incremental warm-up until 60% of HR_max_ • 11-min constant load cycling o facial cooling from minute 5–8	Constant load with fixed duration – 60% HR_max_ – *Light*	• SBFhead and HbO_2_ ↑ during exercise and temporally ↓ with facial cooling • HbO_2_ and tHb changes correlated with the relative changes in SBFhead • HHb and TOI did not change significantly with either exercise or facial cooling • Forehead TOI was not affected by exercise or facial cooling
Rupp et al. (2013)	10 M A: 37 ± 7 year Length: 180 ± 5 cm Mass: 73 ± 7 kg Training status: PL2	Oxymon III, Artinis, the Netherlands IOD: 35 mm Position Fp1 and F3, according to the modified international EEG 10–20 system 780 and 850 nm Continuous wave	(Cycling) 4-h cycling exercise (45% max aerobic power output) 3 consecutive 80-min-bouts (B1, B2, B3), separated by 25-min of neuromuscular function testing	Constant load with fixed duration – 45% of maximal aerobic power output – *Vigorous*	• MVC ↓ after B1, B2 and B3 (-11, -19, and -25%) • RPE ↑ throughout the 3 bouts •△[HbO_2_] and △[tHb] ↑ during B1 reaching a plateau after ∼40 min • [HHb] progressively ↑ during B1 from 20 to 80 min and showed a main effect of bout throughout the protocol •Δ[HHb] ↑ during B2 and B3 sign. reduced compared to B1
Giles et al. (2014)	14 M; 10 F A: 20.21 ± 2.38 year BMI: 22.51 ± 2.72 Training status: PL2	fNIR Imager 1100, fNIR Devices LLC, Potomac, Maryland, United States IOD: 25 mm Position: forehead; bottom of the probe at the Fpz, (international 10–20 system) 730 and 850 nm Continuous wave	(Cycling) 30-min recumbent cycling followed by a 5-min cool down T1: low load (52%HR_max_) T2: moderate load (68%HR_max_) T3: high load (84%HR_max_) Counterbalanced	Constant load with fixed duration – 52%HR_max_ 68%HR_max_ 84%HR_max_ – *Light Moderate vigorous*	• HbO_2,_ HHb and tHb↑ in function of exercise load and time • No change in HbO_2_ during the first minute of pedaling • HbO_2_ = until minute 15, then HbO_2_ ↑ (high and moderate) to low and at minute 18, HbO_2_ (high) to moderate • HHb = until minute 23, then HHb ↑ (high > moderate > low) • tHb = until minute 16, then tHb ↑ (high > moderate > low)
Santos-Concejero et al. (2015)	10 M A: 23.7 ± 4.2 year Length: 170.5 ± 6.3 cm Mass: 54.8 ± 6.3 kg BMI: 18.8 ± 1.3 Fat%: 8.7 ± 0.5% Training status: PL5	NIRO-200X, Hamamatsu, Japan IOD: 30 mm Position: left prefrontal lobe between Fp1 and F3, according to the modified international EEG 10–20 system. 735, 810, and 850 nm Continuous wave	(Running) T1: 5-km time trial T2: MIT T3: constant speed running bouts (running economy determination)	TT – Self-selected speed – *Near to maximal*	5 km TT: • Cerebral oxygenation ↑ over the first half of the trial (↑Δ[HbO_2_] and Δ[HHb]) •Δ[HbO_2_] = in the second half of the trial and Δ[HHb] ↑ until the end • TOI ↓ over the first 1.5 km and then remained stable until completion • tHb: stable for the first half of the test and increased progressively from 3 km until completion • SpO_2_ ↓ at the beginning of the 5-km TT and then remained stable until completion
Pires et al. (2016)	10 M A: 32.9 ± 7.3 year Length: 175.7 ± 5.9 cm Mass: 75.9 ± 9.0 kg Fat%: 10.5 ± 5.2% Training status: PL3	CW6- TechEn, Milford, MA, United States IOD: 45 mm Position: prefrontal lobe at the Fp1 position (international 10–20 system) 690 and 830 nm Continuous wave	(Cycling) T1: Familiarization T2: MIT T3: Self-paced 4 km cycling TT	TT – Self-selected power – *Near to maximal*	•Δ[HbO_2_], Δ[HHb], and Δ[tHb] ↑ up to ∼70% of both MIT and TT4 km, and ↓ afterward •Δ[HbO_2_] was lower in TT4 km than MIT at 20, 30, 40, 50, and 60%, but higher at 100% of the exercise duration • Greater RPE slope in MIT than in TT4 km
Santos-Concejero et al. (2017)	15 M A: 23.7 ± 4.2 year Length: 170.5 ± 6.3 cm Mass: 54.8 ± 6.3 kg Training status: PL5	NIRO-200X, Hamamatsu, Japan IOD: 30 mm Position: left prefrontal lobe between Fp1 and F3, according to the modified international EEG 10–20 system. 735, 810, and 850 nm Continuous wave	(Running) T1: 5-km time trial, T2: MIT T3: Fatigue training test (FTT)	T1: TT Self-selected speed – T2: MIT – T3: FTT 5% faster than 5 km TT pace – *Near to maximal*	FTT: • SpO_2_ ↓ in the first two running bouts, but ↑ in the third bout and remained stable Δ[HbO_2_] • Elevated compared to baseline throughout the fatiguing session •↑ during each bout, and ↓ during the 30 s recovery period until the end • at the end of each repetition ↓ over the course of the trial Δ[HHb] •↓ = negatively correlated with speed at which the test was completed •<↓ Cox between the 1^st^ and 4^th^ and between the 1^st^ and last repetitions in the late fatigue group TOI •↓ throughout the session
Ohyanagi et al. (2018)	6 M, 6 F A: 20.0 year Training status: /	OMM3000; Shimadzu Co., Kyoto, Japan IOD: 30 mm Position: Fpz position of the international 10–20 system 780, 805, and 830 nm Continuous wave	(Cycling) T1: MIT T2: Experimental trial (randomly assigned to upright or supine position): • 4-min pre exercise rest • 4-min warm-up • 20-min at 50% VO_2_max • 15-min PER	Constant load with fixed duration – 50% VO_2_max – *Moderate*	HbO_2:_ •↑ from 3 to 6 min during the 20-min main exercise • Steady from min 6 until the end of exercise SBF: •↑ gradually throughout the exercise • MAP ↑ during the first 3 min of exercise and then ↓ slowly throughout the exercise phase
Radel et al. (2017)	15 M, 7 F A: 21.3 ± 2.1 year Length: 174.7 ± 6.4 cm Mass: 67.0 ± 10.6 kg Peak HR: 186.5 ± 10.9 PAP: 286.1 ± 79.3 Training status: /	Oxymon Mk II, Artinis Medical Systems, Zetten, the Netherlands IOD: 40 mm Position: AF2h and F6h sites of the extended 10–5EEG system 764 and 858 nm Continuous wave	(Cycling) T1: MIT T2 and T3: 60% PAP for 10-min Participants expected one trial to last 60-min	Constant load with fixed duration – 60% PAP – *Near to maximal*	• lower general [HbO_2_] in the first period vs. second- and third periods of time • rdlPFC: [HbO_2_] less elevated in the 60-min than in the 10-min condition • rmPFC: [HbO_2_] higher in the 60-min than in the 10-min condition • Attention was less focused on the exercise trial in the 60-min than in the 10-min condition • RPE =
Tsubaki et al. (2018)	8 M, 4 F A: 21.3 ± 0.7 year Training status: /	OMM3000; Shimadzu Co., Kyoto, Japan IOD: 30 mm Position: Cz position of the International 10–20 System 780, 805, and 830 nm Continuous wave	(Cycling) T1: MIT T2 : Experimental trial • 3-min rest • 20-min at to 50% VO_2peak_ • 15-min PER	Constant load with fixed duration – 50% VO_2peak_ – *Moderate*	• HbO_2_ ↑ between start and the last 5 min of the exercise (R-PFC) • HbO_2_ did not return to pre-exercise levels during the 15-min PER • HbO_2_ higher during the last 5 min of PER than during the pre-exercise rest period • HbO_2_ = between the last 5 min of exercise and 15-min PER. • SBF and MAP ↑ during exercise and ↓ during PER • SBF and MAP = between pre-exercise rest phase and the last 5 min of the 15-min PER.
Hiura et al. (2018)	PET-study 12 M A: 21.1 ± 2 year Training status: / NIRS-study 12 M A: 22.5 ± 2.9 year Training status: /	NIRS : Spectratech, OEG-16, Yokohama, Japan IOD:30 mm Position: Fpz position of the international 10–20 system 770 and 840 nm PET: Discovery PET/CT 710 scanner (GE Healthcare, Milwaukee, WI	(Cycling) 15-min at 30% HR reserve	Constant load with fixed duration – 30% HR reserve – *Light*	•Δ HbO_2_ ↑at Ex2 but did not change at Ex1 •ΔHHb = during exercise • rCBF ↑ at Ex1 but = at Ex2. • PET CO_2_ ↑ at Ex1 and = at Ex2. • MBP ↑ during exercise but ↓ at Ex2 compared with Ex1
Kounalakis and Geladas (2012)	12 M A: 23.4 ± 3.8 year Length: 179 ± 7 cm, Mass: 78.3 ± 6.7 kg, Fat%: 10.4 ± 3.6% Training status: /	NIRS, In Spectra325, Hutchinson Technology Inc., Hutchinson, Minn IOD: / Position: middle of the forehead, just below the hairline 720 and 760 nm	(Cycling) 2 preliminary MIT at 40- and 80 RPM 2 experimental trials: T1: 40 RPM T2: 80 RPM • 8 min warm-up (50% VO_2peak_) • Three 5 s MVC_isometric_ (knee) • 25 min rest on the cycle ergometer • 90 min at 58–60% VO_2peak_	Constant load with fixed duration – 58–60% VO_2peak_ – *Moderate*	• O_2_-cost during 5 min of unloaded pedaling was significantly higher at 80- than at 40 RPM • First 8 min: VO_2_ sign. ↑ (∼250 ml) at 80 than at 40 RPM. • First 8 min: HR = Prefrontal oxygenation: •Δ[HbO_2_]: ↑ from start to end of exercise •Δ[tHb] & Δ[HbO_2_]: lower at 80 vs. 40 RPM at the end of exercise •Δ[HHb]: lower at the end of exercise at 80 RPM •Δ[HHb] = throughout exercise at 40 RPM • SpO_2_: ↓ at 80 than at 40 RPM during exercise
Takehara et al. (2017)	5 M, 8 F A: 21.2 ± 0.6 year Length: 174.7 ± 6.4 cm Mass: 67.0 ± 10.6 kg Peak HR: 186.5 ± 10.9 PAP: 286.1 ± 79.3 Training status: /	OMM3000, Shimadzu Co., Kyoto, Japan IOD: 30 mm Position: Cz position of the International 10–20 System	2 trials: Trial 1: 30% VO_2peak_ Trial 2: 50% VO_2peak_ • 180 s rest period, 10 min continuous cycling exercise at 55 RPM	Constant load with fixed duration – 30% VO_2peak_ 50% VO_2peak_ – *Light Moderate*	30% VO_2peak_ condition • PFC [HbO_2_] ↓ from 30-270s and ↑ from 300 to 600 s • SBF ↑ from 480 to 510 s 50% VO_2peak_ condition • PFC [HbO_2_] ↓ from 30 to 120 s and ↑ from 150 to 600 s • SBF ↑ from 330 to 600s MAP ↑ in both conditions from 60 to 600 s
Ide et al. (1999)	7 M, 7 F A: 20.6 ± 1.4 year Length: 171.0 ± 7.5 cm Mass: 69.3 ± 7.3 kg BMI: 23.4 ± 2.0 Training status: /	NIRO 500, Hamamatsu Photonics, Hamamatsu, Japan. IOD: 45 mm apart, Position: forehead 775, 825, and 850 nm	T1: MIT T2: experimental trial • 10 min rest • 10 min cycling at 30% VO_2_max • 10 min cycling at 60% VO_2_max • 10 min PER	Constant load with fixed duration – 30% VO_2_max 60% VO_2_max – *Light Moderate*	•Δ[HbO_2_]: ↑ in proportion to work rate and reached a maximal level during the first few minutes of exercise • HHb and tHb: ↑ • MCA V_mean:_ ↑ • MAP and HR: ↑ with exercise intensity

*M, man; F, female; A, age; Y, Years; BMI, Body Mass Index; Fat%, body fat; KG, kilogram; Cm, Centimeter; mm, Millimeter; VO2peak, VO_2__max_ Maximal Oxygen Uptake; PL, Performance Level; NIRS, Near Infrared Spectroscopy; IOD, Inter Optode Distance; TTE, Time To Exhaustion; SaO_2_, Arterial Oxygen Saturation; VO_2_, Oxygen Uptake; V_E_, Ventilation; HR, Heart Rate; tHb, Total Hemoglobin; HbO_2_, Oxygenated hemoglobin; HHb, Deoxygenated Hemoglobin; MRI, Magnetic Resonance Imaging; Sec, Second; Min, Minute; Km, Kilometer; H, Hour; BP, Blood Pressure; PFC, Prefrontal Cortex; PMC, Premotor Cortex; m-SMC, Medial Sensori-Motor Cortex; TT, Time-Trial; EEG, Electroencephalography; RPE, Rating of Perceived Exertion; CPT(_RM)_, Constant-Power Test (Respiratory Maneuver); RPM, Rotations per Minute; SBF, Skin Blood Flow; TOI, Tissue Oxygenation Index; MVC, Maximal Voluntary Contraction; MIT, Maximal Incremental Exercise Test; Cox, Cerebral Oxygenation; FTT, Fatigue Training Test; PER, Post-Exercise Rest; MAP, Mean Arterial Pressure; PAP, Peak Aerobic Power; T, Trial.*

**TABLE 5B:** Overview of the results within “maximal incremental exercise (MIT)”.

Study	Sample characteristics (Mean ± SD)	NIRS—devise, Inter Optode Distance (IOD), position, wave specifications	Physical task	Type of task and exercise intensity	Outcome
Bhambhani et al. (2007)	7 M A: 26.7 ± 8.6 year Length: 178 ± 0.6 cm Mass :77.5 ± 9.3 kg BMI: 24.1 ± 2.5 Training status: PL1	MicroRunman, NIM Inc., Philadelphia, PA IOD: 40 mm Position: left pre-frontal lobe, ± 3 cm from midline, just above the supra-orbital ridge	(Cycling) • 2-min rest + baseline measurement • MIT: 30 W/2-min until exhaustion	MIT – ↑ 30 W/2-min	• PET_c__o2_ ↑ systematically until RCT-GEX • After RCT-GEX: PET_c__o2_ ↓ continuously until exhaustion • CBV and Cox ↑ systematically during MIT, slightly beyond the RCT-GEX-intensity, then continuous ↓until exhaustion •↑in Cbv > ↑ in Cox • Sign. difference RCT-GEX, 76.9% and RCT-NIRS, 81.0%, as %VO_2m__ax_
Rupp and Perrey (2008)	13 M A: 24.9 ± 1.5 year Length: 179.3 ± 1.8 cm Mass: 71.1 ± 1.2 kg Training status: PL5	NIRO-300, Hamamatsu Photonics, Japan IOD: 50 mm Position: Fp1 and F3, according to the modified international EEG 10–20 system 775, 810, 850, and 905 nm	(Cycling) • 2-min rest + baseline measurement • 3-min warm-up at 60 W MIT: 30 W/min until exhaustion Pre- and post-MIT: 2 MVICs (knee extensor)	MIT – 60 + 30 W/min	PFC-oxygenation •Δ[tHb] ↑ until VT2 and then stabilized •Δ[HHb] ↑ with the workload after warm-up until exhaustion and finally ↓ toward resting values during recovery •Δ[HbO2] ↑ from warm-up to VT2, then dropped until exhaustion and finally ↑ over resting values during recovery • Exhaustion and finally overshot the resting values during recovery
Timinkul et al. (2008)	10 M A: 21.4 ± 0.6 year Length: 175 ± 1.6 cm Mass: 68 ± 3.6 kg Training status: PL1	NIRO-300, Hamamatsu Photonics, Japan IOD: 50 mm Position: Fp1 and F3, according to the modified international EEG 10–20 system 775, 810, 850, and 905 nm Continuous wave	(Cycling) • 2-min rest + baseline measurement • MIT: 0 + 25 W/3 min until 150 W, 150 + 25 W/min until exhaustion	MIT - 150 + 25 W/min	• HR and VO2 ↑ progressively • Correlation between [Bla] and HHb and HR and HbO2 • TOI response showed a small ↑ (2–7%) or no change (one subject) with ↑ workload. However, before all-out, it began to ↓ gradually • PETCO2 ↑ gradually with ↑ workload and reached its peak, then ↓ gradually until all-out 3 distinct phases 1. Linear oxygenation phase (until blood volume threshold) • HbO_2:_ gradual ↑ in VO_2_ from start till 15 min (100 W). • HHb: small↓ 2. Hyper-oxygenation phase: • HbO_2_: rapid cerebral oxygen intake after 15-min (100 W) • HHb: ± = 3. Desaturation phase: • HbO_2_: oxygenation continuously ↓from respiratory compensation threshold until exhaustion HHb: ↑
Tempest et al. (2014)	13 M, 12 F A: 25.6 ± 3.4 year Length: 174.8 ± 6.8 cm Mass: 71.8 ± 8.3 kg BMI: 23.5 ± 2.2 VO_2p__eak_: 41.8 ± 5.2 ml/kg^–1^*min^–1^ Training status: PL1	Oxymon Mk II, Artinis Medical Systems, Zetten, the Netherlands IOD: 40 mm Position: AF2 h and F6 h sites of the extended 10–5EEG system Continuous wave	(Cycling) • Recumbent cycle ergometer MIT: 20 W/min until exhaustion	MIT – 20 W/min	•ΔHbO_2_ ↑ from below VT to VT and VT to RCP, but remained stable from RCP to End •Δ HHb remained stable from below VT to VT, then ↑ from VT to RCP and RCP to End •Δ tHb increased from below VT, to VT, RCP, and End
Jung et al. (2015)	9 M A: 23–24 year Length: 182 ± 5 cm Mass: 77.6 ± 4.6 kg Fat%: 11.0 ± 2.8% Training status: PL3	Oxymon, Artinis, Zetten, The Netherlands IOD: 25 mm Position: left PFC (Brodmann area 10, MNI coordinates (x/y/z) -40, 50, 0) 858 and 764 nm Continuous wave	(Cycling) • MIT: 1 W/Kg + 1 W/Kg*3-min^–1^ until exhaustion 1 stage = + 1 W/Kg	MIT – 1 W/Kg + 1 W/Kg*3-min^–1^	HbO_2_ • Continuously ↑ from stage 1 to stage 2 and from stage 2 to stage 3 • Leveled between stage 3 and 4 HHb: • No changes
Oussaidene et al. (2015)	Endurance athletes (TR) 13 M A: 26 ± 5 year Length: 178.5 ± 5.8 cm Mass: 70 ± 6 kg Training status: PL3 Untrained (UNT) 11 M A: 24 ± 6 year Length: 179.7 ± 4.9 cm Mass: 77.2 ± 6.1 kg Training status: PL1	Oxymon, Artinis, Zetten, The Netherlands IOD: 50 mm Position: Fp1 and F3, according to the modified international EEG 10–20 system 780 and 857 nm Continuous wave	(Cycling) • 5 min resting period • 3-min warm-up 60 W (UNT), 100 W (TR) • MIT: warm-up workload ↑ with 1 W/3-s until exhaustion • 8-min rest (2 active, 8 passive) • TTE: o 1-min (50 W_max_) o 105% W_max_ until exhaustion	MIT – 60 W or 100 + 1W/3-s TTE – 105%W_max_	HbO_2:_ •ΔHbO2 ↑ from rest to ThCox and ↓ between ThCox and Wmax HHb*:* •ΔHHb ↑ between rest and Wmax in TR and UNT, but only in TR between rest and ThCox tHb*:* •ΔtHb ↑ from rest to W_ThCOx_ and stopped increasing between Th_COx_ and W_max_, in the two groups Only ΔHbO_2_ at Th_Cox_ was higher in TR than in UNT without a difference in W_max_ between groups
Tempest and Parfitt (2016)	High tolerance (HT) 7 M, 7 F A: 20.6 ± 1.4 year Length: 171.0 ± 7.5 cm Mass: 69.3 ± 7.3 kg BMI: 23.4 ± 2.0 Training status: PL2 Low tolerance (LT)	NIRO 200 Hamamatsu Photonics, Hamamatsu, Japan IOD: 40 mm Position: between Fp1 and F3 (left side) and Fp2 and F4 (right side) of the International 10–20 system	(Cycling) • MIT: 20 W/min until exhaustion 70 RPM	MIT – ↑ 20 W/min	• Overall TTE (in seconds) = between HT- and LT-group HbO_2_ •ΔHbO_2_ remained stable from below VT to VT, then ↑ from VT to RCP and from RCP to end in both groups. HHb •ΔHHb remained stable from below VT to VT, that increased from VT to RCP and from RCP to end in both groups tHb •ΔtHb remained stable from below VT to VT, ↑ from VT to RCP and from RCP to end
	7 M, 7 F A: 21.5 ± 3.4 year Length: 173.2 ± 9.1 cm Mass: 68.6 ± 12.8 kg BMI: 22.4 ± 2.7 Training status: PL1				
Tempest et al. (2017)	6 M, 6 F A: 26.2 ± 3.0 year Length: 173.7 ± 7.6 cm Mass: 72.7 ± 9.1 kg Training status: PL1	Oxymon Mk II, Artinis Medical Systems, Zetten, the Netherlands IOD: 38 mm Position: 10/20 international system for electrode placement (the most inferior probes in line with Fpz)	(Cycling) • MIT: 20 W/min until exhaustion. Upright, recumbent and semi-recumbent position.	MIT – 20 W/min	HbO_2_ •ΔHbO_2_↑ from VT to RCP, but remained stable until maximal intensity, and was higher in the left than the right hemisphere. HHb •ΔHHb during upright was similar to recumbent but higher than semi-recumbent cycling •ΔHHb ↑ from VT to RCP and maximal intensities tHb •ΔtHb during upright was similar to semi-recumbent, but higher than recumbent cycling •ΔtHb ↑ from VT, to RCP and maximal intensities
Stevens et al. (2018)	15 M A: 27.8 ± 3.21 year Length: 173.7 ± 7.6 cm BMI: 24.96 ± 3.21 VO_2_max: 41.80 ± 7.69 ml/kg*min^–1^ Training status: PL1	OxiplexTS, ISS, Champaign, Illinois, Unite d States IOD: 20, 25, 30, and 35 mm Position: Fp1 and F2, according to the international EEG 10–20 system 690 and 830 nm	(Cycling) • 2-min pre-exercise rest • 2-min resistance-free cycling at a self-selected cadence 30 W increments every 2 min until exhaustion	MIT – 0 + 30 W/2-min	• HbO_2_ and tHb: little change up to 15% ↑VO_2m__ax_, quadratic increase up to 75% and a small increase above the RCT • HHb: linear trend against exercise intensity COx was unchanged with intensity
Kojima et al. (2020)	4 M, 8 F A: 21.6 ± 0.2 year Length: 162.6 ± 2 cm Mass: 57.3 ± 2.9 kg Training status: /	OMM3000, Shimadzu Co., Kyoto, Japan IOD: 30 mm Position: Cz position of the International 10–20 System	(Cycling) • 4-min pre-exercise rest • 4-min warm-up 20 W increments/min until exhaustion	MIT – 0 + 20 W/min	• HbO_2_ ↑ until RCP and ↓ until exhaustion • HHb ↑ VT until exhaustion but not from rest to VT • tHb ↑ from start to RCP and further to exhaustion • SBF ↑ at VT, RCP and exhaustion compared to rest
Kojima et al. (2021)	24 M A: 20.7 ± 0 year Length: 172.6 ± 5.9 cm Mass: 64.8 ± 9.9 kg Training status: /	LABNIRS, Shimadzu Co., Kyoto, Japan IOD: 30 mm Position: Cz position of the International 10–20 System	(Cycling) • 4-min pre-exercise rest • 4-min warm-up 20 W increments/min until exhaustion	MIT – 0 + 20 W/min	• HbO_2_ ↑ until RCP and ↓ until exhaustion • RCP correlated with the HbO_2_ decreasing point ↑↑ΔHHb, ΔTHb, ΔCBV, and ΔSBF ↑ with load until exhaustion •ΔP_ET_CO_2_ first ↑ with incremental load and then ↓ from 60 to 100% • P_ET_CO_2_ correlated with ΔHHb but not with HbO_2_ and ΔCBV

*M, man; F, female; A, age; Y, Years BMI, Body Mass Index; Fat%, body fat; KG, kilogram; Cm, Centimeter; mm, Millimeter; VO_2_peak, VO_2__max_ Maximal Oxygen Uptake; PL, Performance Level; NIRS, Near Infrared Spectroscopy; IOD, Inter Optode Distance; TTE, Time To Exhaustion; SaO_2_, Arterial Oxygen Saturation; VO_2_, Oxygen Uptake, V_E_, Ventilation; HR, Heart Rate; tHb, Total Hemoglobin; HbO_2_, Oxygenated hemoglobin; HHb, Deoxygenated Hemoglobin; MRI, Magnetic Resonance Imaging; Sec, Second; Min, Minute; Km, Kilometer; H, Hour; BP, Blood Pressure; PFC, Prefrontal Cortex; PMC, Premotor Cortex; m-SMC, Medial Sensori-Motor Cortex; TT, Time-Trial; EEG, Electroencephalography; RPE, Rating of Perceived Exertion; CPT(_RM)_, Constant-Power Test (Respiratory Maneuver); RPM, Rotations per Minute; SBF, Skin Blood Flow; TOI, Tissue Oxygenation Index; MVC, Maximal Voluntary Contraction; MIT, Maximal Incremental Exercise Test; Cox, Cerebral Oxygenation; FTT, Fatigue Training Test; PER, Post-Exercise Rest; MAP, Mean Arterial Pressure; PAP, Peak Aerobic Power; T, Trial; P_ET_CO_2_, End-tidal pressure of CO_2_.*

### Prefrontal Cortex Oxygenation During Maximal Incremental Exercise

Eleven of the included studies evaluated the effects of exercise intensity on cerebral cortex oxygenation using an incremental cycling protocol until exhaustion (Bhambhani et al., 2007; Rupp and Perrey, 2008; Timinkul et al., 2008; Tempest et al., 2014, 2017; Jung et al., 2015; Oussaidene et al., 2015; Tempest and Parfitt, 2016; Tsubaki et al., 2016; Stevens et al., 2018; Kojima et al., 2021). Incremental exercise protocols were characterized by systematic increases in intensity over time. The protocols consisted of fixed increases of 20 W (Tempest et al., 2014, 2017; Jung et al., 2015; Tempest and Parfitt, 2016; Kojima et al., 2020, 2021), 25 W (Timinkul et al., 2008), and 30 W (Rupp and Perrey, 2008) per 1 min, 30 W per 2 min (Bhambhani et al., 2007; Stevens et al., 2018), and 1 W/kg per 3 min (Oussaidene et al., 2015) until exhaustion. All protocols started from very low intensities and ranged from no resistance up to 100 W for the trained athletes. Intensities throughout all the tests were classified as low, moderate, vigorous, and near-maximal to maximal (Garber et al., 2011). From this point, oxygenated (HbO_2_), deoxygenated hemoglobin (HHb), and total hemoglobin (tHb) relate to the PFC unless stated otherwise.

Ten studies reported an increase in [HbO_2_] during the first part of the incremental exercise (Bhambhani et al., 2007; Rupp and Perrey, 2008; Timinkul et al., 2008; Tempest et al., 2014, 2017; Jung et al., 2015; Oussaidene et al., 2015; Stevens et al., 2018; Kojima et al., 2021). Timinkul et al. (2008) described the three distinct phases of prefrontal oxygenation during incremental exercise: (1) the linear-oxygenation phase, where HbO_2_ gradually increases and HHb slightly decreases from the start until the so-called cerebral blood volume threshold (42 ± 3.9% VO_2m__ax_), (2) the hyper-oxygenation phase, where HbO_2_ rapidly increases until the respiratory compensation point (RCP), while HHb remains stable, and (3)the desaturation phase, where HbO_2_ continuously decreases and HHb increases until exhaustion. However, Timinkul et al. (2008) did not express their results as a percentage of VO_2m__ax_. Similarly, Rupp and Perrey (2008) described an increase in [HbO_2_] from a warm-up to the second ventilatory threshold (VT2, 87.0 ± 2.0% VO_2m__ax_) and the cerebral oxygenation threshold (ThCox, 86.0 ± 4.0% VO_2m__ax_ for untrained and 85.0 ± 9.0% VO_2m__ax_ for trained). Similar results were observed for Oussaidene et al. (2015) who reported a decline in HbO_2_ between VT2 or ThCox and exhaustion. These findings were replicated by Kojima et al. (2020, 2021) who reported an increase in [HbO_2_] from the start of exercise until RCP and a subsequent decrease from RCP until exhaustion. Additionally, Kojima et al. (2021) reported a relationship between the point of decline in [HbO_2_] and RCP. Kojima et al. (2021) also reported that end-tidal CO_2_ was decreased by respiratory compensation after RCP and that end-tidal CO_2_ was associated with HHb.

Data in relation to HHb were inconsistent between studies. Timinkul et al. (2008) and Kojima et al. (2020) reported that HHb slightly decreased from the start of the trial until the cerebral blood volume threshold, remained stable until the RCP, and thereafter increased until exhaustion. Rupp and Perrey (2008) and Oussaidene et al. (2015), however, reported that HHb increased from a warm-up until exhaustion. Rupp and Perrey (2008) and Oussaidene et al. (2015) also reported that tHb concentrations increased between rest, VT2, and ThCox, after which it was stabilized until exhaustion. Three studies acknowledged the initial increase in [HbO_2_] from the start of exercise, but instead of a subsequent decrease in [HbO_2_], a steady state was reached (Tempest et al., 2014, 2017; Jung et al., 2015). Two studies reported an increase in [HbO_2_] from the start of an exercise until RCP, after which [HbO_2_] remained stable until exhaustion (Tempest et al., 2014, 2017; Jung et al., 2015). Tempest et al. (2014) reported that [HbO_2_] increases from the value below VT (54.5 ± 4.1% VO_2m__ax_) until RCP (90.2 ± 4.9% VO_2m__ax_) and remains stable from RCP until exhaustion. The authors also showed that [HHb] remains stable between the value below VT and VT (68.2 ± 4.5% VO_2m__a_x) and increases from VT until exhaustion, whereas [tHb] continuously increases from the value below VT until exhaustion. Tempest et al. (2017) found that [HHb] increased from VT until exhaustion, whereas [HbO_2_] remained stable between the start of exercise and VT (67.2 ± 2.9% VO_2m__ax_), increased between VT and RCP (87.9 ± 3.2% VO_2m__ax_), and thereafter remained stable until maximal intensity. Additionally, the results of the study by Tempest et al. (2014, 2017) were supported by Jung et al. (2015) who found a continuous increase in PFC oxygenation from stage 1 to stage 3 and stabilized HbO_2_ concentrations between stages 3 and 4 during a multistage protocol that started at 1 W/kg (stage 1) and increased with 1 W/kg every stage. In contrast to Tempest et al. (2014, 2017) and Jung et al. (2015) found no significant differences in [HHb] between stages/intensities. Finally, two studies reported increases in [HbO_2_] from RCP until exhaustion. Tempest and Parfitt (2016) measured prefrontal oxygenation during a maximal incremental test (MIT) between groups with high- and low-self-reported tolerance to exercise. The data indicated that [HbO_2_] remains stable between the value below VT and VT (high = 49.8 ± 4.8% VO_2m__ax,_ low = 54.6 ± 5.0% VO_2m__ax_) and increased between VT and RCP VT (high = 76.3 ± 5.1% VO_2m__ax_, low = 84.1 ± 5.9% VO_2m__ax_) and from RCP to exhaustion. According to Tempest et al. (2014, 2017), [HHb] and [tHb] remained stable from the value below VT to VT, increased from VT to RCP, and also increased from RCP to exhaustion in both tolerance groups. In contrast, Stevens et al. (2018) reported a small increase in [HbO_2_], [HHb], and [tHb] up to 15% VO_2m__ax_, followed by a quadratic increase up to 75% VO_2m__ax_ and a smaller increase above the value of RCT. Importantly, [HbO_2_], [HHb], [tHb] increases were most prominent at workloads near RCP (Stevens et al., 2018). A visual representation of HbO_2_ and HHb during MIT can be found in Table 6.

**TABLE 6:** Summary of finding on the evolution of HbO_2_ and HHb during MIT.

	HbO_2_			HHb		
Study	Start—VT	VT—RCP	RCP—exhaustion	Start—VT	VT—RCP	RCP—exhaustion
Bhambhani et al. (2007)	↑	↑	↓	NA	NA	NA
Timinkul et al. (2008)	↑	↑	↓	↓	**=**	↑
Tempest et al. (2014)	↑	↑	**=**	**=**	↑	↑
Jung et al. (2015)	↑	↑	**=**	**=**	**=**	**=**
Oussaidene et al. (2015)	↑	↑	↓	↑	↑	↑
Tempest and Parfitt (2016)	**=**	↑	↑	**=**	↑	↑
Tempest et al. (2017)	=	↑	**=**	**=**	↑	↑
Stevens et al. (2018)	↑	↑	↑	↑	↑	↑
Kojima et al. (2020)	↑	↑	↓	↓	**=**	↑
Kojima et al. (2021)	↑	↑	↓	↑	↑	↑

*VT, Ventilatory threshold (50–70%VO_2m__ax/peak_); RCP, Respiratory compensation point (80–90% VO_2m__ax/peak_); Exhaustion (100% VO_2m__ax/peak_).*

### Prefrontal Cortex Oxygenation During Whole-Body Endurance Tasks

Seventeen studies measured TTE, TT, a constant load with fixed intensity- and adaptive walking/running-exercise protocols. Eleven of the included studies assessed PFC oxygenation during whole-body endurance protocols with a constant intensity and fixed duration (Ide et al., 1999; Fumoto et al., 2010; Miyazawa et al., 2013; Rupp et al., 2013; Giles et al., 2014; Tsubaki et al., 2016, 2018; Radel et al., 2017; Takehara et al., 2017; Hiura et al., 2018; Ohyanagi et al., 2018). In these studies, participants were asked to complete an endurance cycling task at a certain predetermined intensity (e.g., %HRmax and %VO_2p__eak_) for a predetermined time. We classify these studies according to the following intensities: (1) light to moderate, (2) vigorous, (3) near-to-maximal, and (4) supramaximal.

*Light to moderate intensity*: two studies assessed PFC during 10 min of cycling at 30 and 50% VO_2p__eak_ (Takehara et al., 2017) and 30 and 60% VO_2m__ax_ (Ide et al., 1999). Ide et al. (1999) found an increase in [HbO_2_], [HHb], and [tHb] in proportion to work rate, at the intensities of 30 and 60% VO_2m__ax_. Increases were significantly higher at 60% VO_2m__ax_, and [HbO_2_] reached a maximal level during the first few minutes of recovery. These data were replicated by Takehara et al. (2017) who reported an increase in [HbO_2_] between 300 and 600 s, which was higher at 50% VO_2p__eak_ than 30% VO_2p__eak,_ but reported a decrease in [HbO_2_] between 30 and 300 s. During a 4-min facial cooling intervention to reduce prefrontal skin blood flow and [HbO_2_], Miyazawa et al. (2013) reported that prefrontal [HbO_2_] increased during an 11-min cycling exercise at 60% HRmax (light intensity). The authors reported that neither the light-intensity cycling exercise nor the facial cooling intervention had an influence on [HHb]. The changes in [tHb] were similar to the changes in [HbO_2_] and were related to prefrontal skin blood flow (Miyazawa et al., 2013). In a 15-min cycling task at 30% HR reserve (light intensity), Hiura et al. (2018) reported that [HbO_2_] remained stable during the first 5 min but increased during the last 2 min of the trial and that HHb remained unchanged during the entirety. Hiura et al. (2018) also monitored the prefrontal blood flow and partial pressure of CO_2_ (PCO_2_) and reported that cerebral blood flow (CBF) and PCO_2_ increased during the first 5 min and remained unchanged during the last 2 min. The results of the study by Ide et al. (1999) were replicated by Fumoto et al. (2010) who found a gradual increase in [HbO_2_] during a 15-min cycling task at an intensity equaling 12–13 on the RPE scale (Borg, 1998) (moderate intensity), after which [HbO_2_] reached a steady state until the end. Only a small decrease in [HHb] was found throughout the trial (Fumoto et al., 2010). A gradual increase in [HbO_2_] during moderate-intensity exercise was supported by Tsubaki et al. (2018) who reported that [HbO_2_] continually increased in right PFC during a 20-min cycling exercise trial at an intensity of 50% VO_2p__eak_. Prefrontal skin blood flow also increased over the time of the trial and decreased again during the postexercise rest (Tsubaki et al., 2018). In a 20-min moderate-intensity (50% VO_2m__ax_) cycling trial, an increase in [HbO_2_] was found between 3 and 6 min after which HbO_2_ levels remained stable until the end (Ohyanagi et al., 2018). Within this research, prefrontal skin blood flow increased gradually throughout the trial, supporting the findings of Miyazawa et al. (2013) and Tsubaki et al. (2018). Finally, in two separate 90-min moderate-intensity cycling tests at 58–60% VO_2p__eak_, at the cadence of 40 and 80 RPM, it was found that at the end of the exercise, [tHb] and [HbO_2_] values were lower at 80 RPM than at 40 RPM, and [HHb] was lower at 80 RPM but remained unchanged at 40 RPM.

Suzuki et al. (2004) measured PFC after performing a 90-s locomotor task at three different speeds (3, 6, and 9 km/h), each with a 30-s rest. Before the start of the task, [HbO_2_] and [tHb] increased, which was most prominent at 9 km/h, and peaked prior to the treadmill speed steadying. After reaching a constant speed, [HbO_2_] and [tHb] decreased and returned to baseline, or lower than baseline, during the rest of the task. After stopping locomotion, temporal drops in [HbO_2_] were seen before returning to baseline. PFC activation was greater, and HbO_2_ was higher at 9 km/h than 3 and 6 km/h. No differences were shown between and 3 and 6 km/h (Suzuki et al., 2004).

*Vigorous intensity*: two studies examined the effects of vigorous cycling exercise on prefrontal oxygenation (Rupp et al., 2013; Giles et al., 2014). Rupp et al. (2013) asked participants to perform three consecutive 80-min cycling trials at 45% peak aerobic power output, each separated by 25 min of neuromuscular function testing. An increase in [HbO_2_] and [tHb] was found in trial 1, reaching a plateau after approximately 40 min, whereas [HHb] progressively increased between 20 and 80 min in trials 1, 2, and 3. [HHb] was significantly lower in trials 2 and 3 than trial 1. In a sample of regular exercisers, Giles et al. (2014) found that [HbO_2_], [HHb], and [tHb] increased as a result of exercise intensity. Participants completed three separate 30-min cycling trials at 52% (low intensity), 68% (moderate), and 84% (vigorous) HRmax, respectively (Giles et al., 2014). Similar HbO_2_ values were found during the first 15 min among exercise intensities. After 18 min, HbO_2_ was significantly higher at a moderate and high intensity than low intensity, and significantly higher at a vigorous than moderate intensity. Higher values of [HHb] were reported as a result of exercise intensity with higher values of [HHb] at a vigorous than moderate intensity, which was also higher than a low intensity (Giles et al., 2014). HHb values were steady until min 12 after which the values increased over time to min 23. From this point, HHb increased according to exercise intensity, with greater increases in [HHb] at a vigorous than moderate intensity, which in turn was higher than low intensity. [tHb] increased with each minute from the start and was similar between intensities until min 16, after which [tHb] increased between all intensities.

*Near-to-maximal intensity*: Two studies reported similar results in PFC during a 5-km running TT. Billaut et al. (2010) reported increased prefrontal [HbO_2_], [HHb], [tHb], and CBV values from the start until a 2.5-km point, from which these values remained constant until km 4.5 and displayed evidence of deoxygenation in the last 0.5 km. Regional blood flow remained stable throughout this. Santos-Concejero et al. (2015) described increases in [HbO_2_] and [HHb] over the first half of the trial, steady HbO_2_ values in the second half of the trial, and further increases in [HHb] until completion. Additionally, TOI reduced over the first 1.5 km, after which it remained stable until the end. However, in contrast to Billaut et al. (2010), [tHb] remained stable for the first half of the trial and increased progressively from 3 km until completion. Pires et al. (2016), used a 4-km cycling TT and found that prefrontal [HbO_2_], [HHb], and [tHb] increased until 70% of the TT and decreased upon completion. Pires et al. (2016) also assessed prefrontal oxygenation during a preliminary MIT (100 W + 25 W/min, until exhaustion), where prefrontal [HbO_2_], [HHb], and [tHb] increased until 70% of the exercise, and thereafter decreased. Santos-Concejero et al. (2017) used a fatigue training test consisting of continuous 1-km repeated running trials, with 30-s recovery until exhaustion, at a pace 5% faster than their 5-km running TT (completed 2 days earlier). During the test, △[HbO_2_] was elevated compared to baseline throughout the test, decreased at the end of each repetition, and progressively decreased in the first, fifth, and final repetition. Δ[HHb] increased over the course of the test with increases during each running bout and decreases during each 30-s recovery period until completion. A decline in cerebral oxygen was negatively associated with the speed at which the test was completed. Radel et al. (2017) examined hemodynamic changes when coping with the expectation of prolonged exercise. Participants were told that they would perform a cycling trial at an intensity equivalent to 60% peak aerobic power and that it would take 10- or 60-min according to the condition. In both experimental sessions, the trial was stopped after 10 min. A main effect of time was found, whereby [HbO_2_] was lower in the first period than in the second and third. Compared to the 10-min condition, in the 60-min condition, smaller [HbO_2_] elevations at the right dorsomedial PFC and higher [HbO_2_] elevations at the right dorsomedial PFC were reported. No differences in RPE were found between conditions.

*Supramaximal intensity*: Shibuya et al. (2004) studied the effect of a supramaximal TTE performance at 120% of participants’ VO_2p__eak_ on cerebral oxygenation. [HbO_2_] increased during the first 30 s of exercise, after which [HbO_2_] and [tHb] gradually decreased over time, resulting in significantly decreased values from the pre-exercise level. [HHb] remained constant throughout the test.

## Discussion

In this review, we outlined the current knowledge on the development of PFC oxygenation during whole-body endurance exercise through fNIRS. Research indicates that during MIT, HbO_2_ increases until RCP, after which it decreases until exhaustion or remains in a steady state. Regarding submaximal exercise, prefrontal oxygenation increases during light, moderate, and vigorous intensity workloads but reaches a steady state over time. According to the findings using MIT at a near-maximal intensity, prefrontal oxygenation cannot be maintained, and deoxygenation occurs at PFC. RCP appears to act as a ThCox, with the occurrence of the deoxygenation of PFC when RCP exceeds. The findings presented in this review show no evidence for an increase in cerebral energy metabolism to be responsible for the deoxygenation of the PFC but instead indicate that an increase in respiratory ventilation in reaction exercise-induced hypocapnia results in a decrease in CBF and thus [tHb], [HbO_2_], and [HHb].

Quality assessment was scored as moderate to strong in most studies, with only three studies being scored as weak (Takehara et al., 2017; Ohyanagi et al., 2018; Kojima et al., 2020). Two major limitations regarding the quality of studies were: (1) the method of participants’ selection or source information variables being insufficiently described, whereby participants are often described as “healthy volunteers,” and (2) insufficient data being presented to assess if the sample size was appropriate. The majority (74%) of the studies used observational study design and were therefore exempted from the subquestions E, F, G, and L of the quality assessment.

### Near-IR Spectroscopy Methodology

As part of the inclusion criteria, all studies used NIRS to investigate PFC oxygenation. With this technique, near-IR light (NIR) is emitted through the scalp up to the neuronal tissue, where the light is either absorbed or scattered off. NIR with wavelengths between the optical window of 700 and 900 nm pass through most biological tissues, including bone because there is low absorption and scattering of photons (Ekkekakis, 2009). A body of research has shown that NIRS with wavelengths around 760 nm has an absorption peak within HHb, whereas in HbO_2_ this is the case at 830 nm, making HHb and HbO_2_ distinguishable from each other (Ekkekakis, 2009). In terms of NIRS device positioning, 23 studies used the international EEG 10–20 or 10–5 system, which is recognized to describe the placement of the scalp electrodes. This is in accordance with the methodological review of Herold et al. (2018) who stated that the international EEG 10–20/10–5 system is the most common and practical strategy for optode placement and thereby ensures that the region of interest is targeted. Next to the optode placement, the IOD is an important factor as it determines the depth of the NIRS measurement. Of the included studies, 16 applied an IOD of between 30 and 40 mm, which is appropriate for adults (Leon-Carrion and Leon-Dominguez, 2012; Herold et al., 2018). However, two studies used an IOD of 25 mm (Giles et al., 2014; Jung et al., 2015), which has been shown to include gray matter into the sample volume, and eight studies used an IOD of between 45 and 50 mm (Ide et al., 1999; Shibuya et al., 2004; Rupp and Perrey, 2008; Timinkul et al., 2008; Fumoto et al., 2010; Keramidas et al., 2011; Oussaidene et al., 2015; Pires et al., 2016), where the contribution of extracranial tissue is negligible (Leon-Carrion and Leon-Dominguez, 2012). Another effect of using a short channel (IOD ≤ 25 mm) is that NIRS signals are influenced by skin blood flow and CBF due to a more superficial measurement (Matsukawa et al., 2015). A consensus is needed for the studies that use NIRS devices to allow appropriate comparisons between studies. The guidelines for the usage of NIRS can be found in Herold et al. (2018).

### Prefrontal Cortex Oxygenation During Incremental Exercise (Maximal Incremental Test)

It was hypothesized that prefrontal oxygenation would increase during submaximal exercise and subsequently decrease at near-maximal intensities. The results of this systematic review largely support this hypothesis but also reveal some new insights into central regulation during endurance performance. Rooks et al. (2010) concluded that cerebral oxygenation increases from low-to-vigorous intensities after which it reaches a plateau or declines toward baseline at near-to-maximal intensities. Rooks et al. (2010) focused on cerebral oxygenation in general, this review specifically focused on PFC and how oxygenation in this brain region evolves during whole-body endurance exercise. About 9 of 10 MIT studies found an increase in [HbO_2_] during the first part of the incremental exercise. This initial increase can be explained by peripheral hemodynamic changes, such as increased cardiac output and metabolic demand in the function of the start of locomotion, and a gradual increase of intensity. Only in Tempest and Parfitt (2016) did [HbO_2_] remain unchanged from the start until VT. All of the included studies reported an increase in [HbO_2_] from VT until RCP, after which prefrontal oxygenation decreased (Bhambhani et al., 2007; Rupp and Perrey, 2008; Timinkul et al., 2008; Oussaidene et al., 2015; Kojima et al., 2020, 2021) or reached a plateau (Tempest et al., 2014; Jung et al., 2015; Tempest and Parfitt, 2016), supporting previous research (Rooks et al., 2010). Additionally, Kojima et al. (2021) reported that the point at which [HbO_2_] declined was correlated with RCP. On the other hand, two studies found a further increase in [HbO_2_] (Tempest and Parfitt, 2016; Stevens et al., 2018). In comparison with a steep increase from VT until RCP, Tempest and Parfitt (2016), did not declare or provide details on the degree of the increase in [HbO_2_], whereas Stevens et al. (2018) reported only a small increase in [HbO_2_] from RCP until exhaustion. These results together with the other seven studies show that RCP is a crucial point for prefrontal oxygenation as after this point prefrontal oxygenation endures significant changes. RCP can be defined as the point at which arterial PCO_2_ starts to decline during strenuous exercise and can be interpreted as a ventilatory response to maintain the acid-base balance by increasing ventilation (Rausch et al., 1991; van den Aardweg and de Groot, 2015).

Two studies (Timinkul et al., 2008; Kojima et al., 2021) measured end-tidal CO_2_ pressure during MIT. Timinkul et al. (2008) found that end-tidal CO_2_ pressure gradually increased with intensity and reached its peak at RCP, after which it gradually decreased until exhaustion. These results were supported by Kojima et al. (2021) who found an increase in end-tidal CO_2_ pressure with incremental load and a subsequent decrease from 60% to 100% of the MIT. This decrease was also correlated with [HHb] and PCO_2_. Research has shown that PCO_2_ has a direct impact on CBF (Lassen, 1959; Poulin et al., 1996; Yoon et al., 2012). The blood-brain barrier is permeable to CO_2_ but retains [H ^+^ ] and [HCO_3_^–^] ions, making CO_2_ an important respiratory stimulant to the central chemoreceptors (Ainslie et al., 2007; Galán-Rioja et al., 2020). The brain requires a constant CBF within a narrow range of 60 and 150 mmHg despite changes in mean arterial pressure (MAP) (Ogoh and Ainslie, 2009). This regulatory mechanism is called cerebral autoregulation (Ogoh and Ainslie, 2009). Hyperventilation caused by intense exercise subsequently results in a drop in PCO_2._ The state of low PCO_2_ in the arterial blood, also referred to as hypocapnia, causes cerebral vasoconstriction, a cerebral autoregulatory mechanism, which results in a decrease in CBF (Ogoh and Ainslie, 2009). Nybo and Rasmussen (2007) stated that when CBF and cerebral oxygenation fall below a critical level, the motor output cannot be maintained. These results have been demonstrated in several studies that prolonged aerobic exercise was improved by increasing cerebral oxygenation through additional inspired O_2_ levels (Nielsen et al., 1999; Subudhi et al., 2007). Besides the association between [HbO_2_] and RCP, Kojima et al. (2021) found that end-tidal CO_2_ decreases after RCP. However, no correlation between end-tidal CO_2_, CBF, and cerebral O_2_ exchange was reported. Kojima et al. (2021) suggests that a decrease in [HbO_2_] and an increase in [HHb] before exhaustion during MIT are related to cerebral O_2_ metabolism by a neural activity increase. While this is an interesting suggestion, it must be cautioned as cerebral blood volume and cerebral oxygen exchange are only the estimated values. Even though NIRS is not capable of measuring blood flow, a decrease in CBF through PCO_2_ and cerebral autoregulation is a promising hypothesis to help explain changes in prefrontal oxygenation at RCP. However, there is a lack of research to objectively assess both cerebral oxygenation and CBF simultaneously. Studies combining NIRS with CBF velocity measurement techniques, such as transcranial doppler (TCD) ultrasonography and PET, could help elucidate the mediators of prefrontal oxygenation during exercise and the role of RCP as a ThCox.

### Prefrontal Cortex Oxygenation During Whole-Body Endurance Tasks

This study is the first review to collect empirical evidence on PFC oxygenation during non-incremental whole-body endurance exercise. It was hypothesized that prefrontal oxygenation would increase during prolonged submaximal exercise until a steady state is reached. Additionally, we hypothesized that during near-maximal intensities PFC oxygenation cannot be maintained, resulting in a decrease near the exhaustion or end of the exercise. Maximal incremental studies have shown that PFC oxygenation is sensitive to exercise intensity. As a result, we have subdivided this section into light-to-moderate, vigorous, and near-to-maximal intensity.

#### Light-to-Moderate Intensity

Three low-intensity protocol studies found an increase in [HbO_2_] from the start to the end of exercise (Ide et al., 1999; Miyazawa et al., 2013; Takehara et al., 2017). Although in the study of Takehara et al. (2017) an initial decrease in [HbO_2_] was found between the start and 3 min into the cycling trial, this can be explained given that O_2_-supply does not match the O_2_-demand at the beginning of exercise (McArdle et al., 2010). Miyazawa et al. (2013) supported these findings and found a correlation between TOI, HbO_2_, and tHb with prefrontal skin blood flow. This triggers the question of whether HbO_2_ was measured at PFC and not superficially at the skin of the scalp, as vasoconstriction through cooling results in a decreased skin blood flow. Alternatively, HHb was not correlated with skin blood flow as HHb was not affected by the face cooling intervention, nor by an 11-min cycling task. Importantly, Miyazawa et al. (2013) implemented a 4-min incremental warm-up until reaching the intensity of 60% HRmax. This can help explain why HHb was not changed throughout the 11-min cycling exercise as several studies have pointed out that HHb is correlated with exercise intensity (Rupp and Perrey, 2008; Stevens et al., 2018; Kojima et al., 2021). Only one study objectively measured prefrontal blood flow and end-tidal CO_2_ during light-intensity exercise (Hiura et al., 2018). The authors reported that the prefrontal CBF and end-tidal pressure of CO_2_ initially increased but remained unchanged during the last few minutes of exercise. Similarly, the initial decrease in [HbO_2_] was simultaneously associated with a steep increase in end-tidal CO_2,_ which can be explained though O_2_ deficit at the start of the exercise. However, it is important to note that this study used both NIRS and PET to measure oxygenation and CBF, respectively, in two different samples. It is therefore unclear whether the results would be similar in the case of measurement at the same time point in the same person. Future research is needed to examine whether these results will be the same when oxygenation and CBF are measured in the same sample.

Regarding moderate intensity endurance tasks, prefrontal oxygenation gradually increases in [HbO_2_] over time until a steady state is reached. Ohyanagi et al. (2018) only found an increase in [HbO_2_] between 3 and 6 min during a 20-min moderate-intensity cycling exercise, after which [HbO_2_] stabilized to completion. Interestingly, Ohyanagi et al. (2018) and Tsubaki et al. (2018) both measured skin blood flow and reported that skin blood flow increased over the time of the exercise and decreased again during the postexercise rest, supporting the results of Miyazawa et al. (2013).

A gradual increase in [HbO_2_] over time is present both at the light and moderate intensities and supports the results of the abovementioned incremental exercise research. In MIT, prefrontal-HbO_2_ levels increase to RCP, which persist in above moderate intensities. This indicates that at light-intensity and moderate-intensity exercise, PFC oxygenation increases gradually until a steady state is reached. However, it is not clear whether the increased prefrontal oxygenation is the result of an increase in cerebral O_2_ metabolism and neuronal activity. An increase in skin blood flow, through elevated MAP, seems to be correlated with increases in [HbO_2_]. The changes measured by NIRS are the result of changes that occur throughout the volume of tissue traversed by the NIR light. The signal will therefore invariably contain a superficial interference from both emitter and receiver optodes. The inclusion of short-separation channels into the NIRS optode template could reduce superficial contamination through skin blood flow, which is influenceable by systemic changes (Gagnon et al., 2014). Gagnon et al. (2014) found a 33% reduction in signal noise for HbO_2_ with the inclusion of one short channel and a 59% noise reduction when two short channels, one at the emitter and one at the detector, in comparison with the standard method. The role of an increased cerebral O_2_ metabolism through neuronal activity can, however, not be ruled out. Suzuki et al. (2004) stated that PFC might be involved in controlling locomotion to adapt to the increasing speed in the acceleration phases during walking and running. Before the start of locomotion, an increase in [HbO_2_] and [tHb] was found, especially during the 9 km/h trial, indicating a neuronal activation at PFC.

#### Vigorous Intensity

Two studies investigated the effects of vigorous cycling exercise on PFC oxygenation (Rupp et al., 2013; Giles et al., 2014). The results showed that prefrontal oxygenation [HbO_2_] increased until reaching a steady state, which is similar during light-to-moderate intensities. Vigorous intensity ends at the verge of RCP but is still located below this threshold. In Rupp et al. (2013) and Giles et al. (2014), HHb remained unaffected during the first few minutes of exercise after which it progressively increases. Interestingly, both HbO_2_ and tHb start to increase from the onset of an endurance task. However, CBF is not directly measured but can be interpreted as an estimate of tHb and HbO_2_. This illustrates that there is an increase in O_2_ supply to the PFC during the initiation of vigorous-intensity exercise. The continuous gradual increase of HbO_2_, tHb, and, therefore, CBF, during 84% HRmax (vigorous intensity), is likely the result of RCP occurring between 85 and 91% HRmax. Given that the targeted workload of 84% HRmax is below this range, it may have resulted in being insufficient to meet RCP and thus a decrease in HbO_2_ and tHb. This confirms our hypothesis that PFC increases during prolonged submaximal exercise until a steady state is reached.

#### Near-to-Maximal Intensity

Six studies assessed prefrontal oxygenation at near-to-maximal intensities. Incremental exercise studies have suggested that PFC oxygenation cannot be maintained and occurs in PFC. The tipping point of this deoxygenating phase is RCP. Five studies confirm this hypothesis during both running and cycling exercises. Billaut et al. (2010) and Pires et al. (2016) showed that prefrontal oxygenation could not be maintained throughout the trial, whereas in Santos-Concejero et al. (2015) HbO_2_ and tHb increased from km 2.5 until the end. Despite the presence of identical TT running trials, Santos-Concejero et al. (2015) included elite Kenyan runners who underwent prenatal exposure to high altitude and high physical activity levels during childhood, which may have facilitated the maintenance of cerebral oxygenation. Prefrontal deoxygenation shown in Billaut et al. (2010) and Pires et al. (2016) did not result in a decrease in the motor output. These results may indicate that the duration of the TT task is insufficient for PFC oxygenation to influence the motor output and that deoxygenation remains within a range that does not hinder strenuous performance. However, these findings do not discount the suggestion that pacing strategies may influence the cerebral function metabolic status. Moreover, an initial increase in blood flow along with HbO_2_ and HHb was thought to be the result of an increase in cardiac output. Because the near-to-maximal intensities exceed RCP, cerebral vasoconstriction could have led to prefrontal deoxygenation. This statement could, however, be questioned as to the deoxygenation in Santos-Concejero et al. (2015) was characterized by an increase in tHb through elevated HHb levels and a decrease in HbO_2._ Similarly, Santos-Concejero et al. (2017) found that during a fatigue training test, △[HbO_2_] that was measured at the end of each running repetition was decreased over the course of the trial. The decrease in HbO_2_ occurred simultaneously with increases in HHb over the course of the session with increases during each running bout, and a subsequent decrease during each 30-s recovery period until the end. It was stated that the speed at which participants performed was significantly faster than their expected speed at RCP, which is supported by a high lactate observation. A major limitation to this study, however, is that tHb, an indicator for CBF, is not measured in this study. Therefore, it is likely that hyperventilation-induced hypocapnia after RCP leads to deoxygenation. This study also highlights that high-intensity interval training (HIIT) results in a decrease in prefrontal oxygenation. In relation to prolonged endurance exercise, this could suggest that sequential intermediate near-to-maximal intensity efforts (e.g., repeated breakaways in cycling races) can result in a decrease in prefrontal oxygenation.

Regarding supramaximal exercise, Shibuya et al. (2004) examined TTE performance at 120% and showed that [HbO_2_] increased during the first 30 s, after which [HbO_2_] and [tHb] gradually decreased and were significantly lower than pre-exercise. [HHb] remained unchanged throughout the trial. The initial increase in [HbO_2_] and [tHb] is likely to be the result of a sudden increase in blood pressure through the start of locomotion against high resistance. On the other hand, the decrease in [HbO_2_] and [tHb] can be linked with increasing end-tidal CO_2_ concentrations, which result in cerebral vasoconstriction through hyperventilation, and in turn, lead to a decrease in CBF and thus in tHb and HbO_2_. Shibuya et al. (2004) proposed a potential role for cerebral fatigue as a result of the changes in oxygenation. However, no study has provided evidence to support this hypothesis. This is the only study that has assessed PFC oxygenation during supramaximal exercise, indicating a need for further research measuring cerebral oxygenation during prolonged supramaximal exercise.

Radel et al. (2017) investigated the hemodynamic changes that arise when coping with the expectation of prolonged exercise and found that [HbO_2_] increased over a 10-min cycling task, which is in accordance with the study of Rooks et al. (2010). When participants were expected to cycle for 60 min, the authors reported that [HbO_2_] elevations at the right dorsolateral PFC were diminished and [HbO_2_] elevations at the right dorsomedial PFC were higher. No differences in RPE between 10 and 60 min were found. The dorsolateral PFC is a representative region for the central executive network, whereas the dorsomedial PFC is a representative region for the default mode network (Fox et al., 2009). This highlights that the brain attempts to save mental resources by providing less activation of brain regions (dorsal PFC) associated with mental effort (the central executive network) and more toward those related to the resting activity (the default mode network) (Shibuya, 2011; Perrey and Besson, 2018). This study also indicates that psychological factors influence PFC oxygenation, suggesting that psychological interventions could positively influence PFC oxygenation and in turn improve endurance performance. Further, nutrition has also been shown to have a facilitating effect on PFC oxygenation. Decroix et al. (2018) reported an elevation in prefrontal oxygenation without having an impact on muscular oxygenation during a 20-min steady-state cycling exercise at 45% VO_2m__ax_ (moderate) after the supplementation of cocoa flavanols. Given that mental fatigue negatively alters endurance performance (Marcora et al., 2009; Martin et al., 2018; Pageaux and Lepers, 2018), these findings are therefore an important factor for an examination within mental fatigue and highlight that PFC is involved in proactive behavior and goal-directed exercises (Pires et al., 2018).

### Respiratory Compensation Point as a Cerebral Oxygenation Threshold

Research on PFC oxygenation within dynamic whole-body endurance performance throughout the entire spectrum of intensities (low, moderate, vigorous, near-to-maximal, and supramaximal intensities) is in accordance with research examining maximal incremental exercise. Our systematic review has found strong evidence that RCP is an important ThCox. Collectively, research indicates that PFC oxygenation increases throughout intensities below the RCP threshold and reaches a steady state during continuous sub-RCP workload tasks. However, during prolonged exercise at the intensities that exceed RCP, the increase or preservation of prefrontal HbO_2_ cannot be maintained and the deoxygenation of PFC takes place. The findings presented in this review show no evidence that an increase in cerebral energy metabolism is responsible for the deoxygenation of PFC and limiting factors of endurance performance. When RCP is reached during exercise, an increase in respiratory ventilation results in hypocapnia. Cerebral autoregulation reacts to hypocapnia by causing cerebral vasoconstriction, resulting in a decrease in CBF and thus in [tHb], [HbO_2_], and [HHb] (Ogoh and Ainslie, 2009).

### Limitations and Future Research

Although this systematic review has provided novel insights into the influence of PFC oxygenation on whole-body endurance exercise, there are some limitations that need to be considered. Firstly, there is a need for a consensus and transparency in reporting the results within whole-body endurance exercise research. RCP acts as a tipping point/threshold for PFC oxygenation. However, this threshold was not consistently determined or referenced in every study. To ensure the adequate comparison between studies and to classify exercise intensity, we calculated VO_2_ and % of VO_2m__ax_ of the described datapoints and the metabolic equivalent of task (MET). To facilitate a comparison between studies in future research, each threshold or discussed data point should be referred to as a percentage of VO_2m__ax/peak_. Furthermore, concerning the reporting of the results, the two factors that are overlooked in most of the included studies are MAP and the training status of the subjects. MAP is closely related to oxygenation and (cerebral) blood flow and can, therefore, give interesting insights into the discussion of the results. However, recently, Castle-Kirszbaum et al. (2021) released a systematic review on a cardio-cerebral coupling and indicated that the current literature is insufficiently robust to confirm an independent relationship between the cardiac output and CBF. Regarding the training status, future research within this field should address participant characteristics related to the performance level as described by De Pauw et al. (2013) and Decroix et al. (2016) for male and female subjects, respectively. Secondly, future research should include objective measures for CBF (e.g., PET and TCD) simultaneously with NIRS to confirm the hypothesis that exceeding RCP results in cerebral vasoconstriction that in turn decreases PFC oxygenation. Moreover, a short-separation channel (8 mm) (Herold et al., 2018) needs to be included within the NIRS setup to determine whether changes in cerebral oxygenation are not the result of changes in local skin blood flow. However, there is a lack of consensus for the fNIRS application (optode placement, IOD, etc.), which complicates comparisons between studies (see Herold et al., 2018 for guidelines).

In this systematic review, only 6 of 28 studies assessed laterality. In future research, PFC laterality is an important factor given that the left and right PFC play different roles and may influence decision-making during endurance exercise. However, given a low number of studies to assess laterality, we believe that it was inappropriate to make comparisons between all the studies. Future research should, therefore, endeavor to examine PFC laterality to provide a greater understanding of how this may influence endurance performance. Another limitation is that the environmental conditions were rarely mentioned. Given that ambient temperature and relative humidity influence endurance performance and can facilitate vasoconstriction or vasodilatation, it is likely that these could influence NIRS parameters. Further research should examine if the deoxygenation of PFC determines whether one stops or prolongs during an endurance task and if the interventions that postpone PFC deoxygenation could result in improved performances. Recently, the first study on this subject has been published (Dallaway et al., 2021). The authors reported that brain endurance training resulted in improved performance on muscular endurance handgrip tasks, which might occur with higher prefrontal oxygenation. This training-induced increase in prefrontal oxygenation was accompanied by a reduced mental effort during the physical task, making oxygenation a potential countermeasure for mental fatigue. These preliminary data are promising, and a need exists for more research to replicate this study design on whole-body endurance exercise tasks.

## Conclusion

To conclude, this systematic review provides a detailed overview of how cerebral oxygenation at PFC reacts to various exercise intensities. As hypothesized, we found that PFC oxygenation increases at low, moderate, and vigorous intensities and decreases at near-to-maximal and supramaximal intensities. Moreover, a steady state could be reached at the intensities below RCP. RCP can also be identified as an important ThCox given that PFC oxygenation cannot be maintained and decreases until the cessation of whole-body endurance exercise at this point. The proposed mechanism behind this is that through an increase in ventilation, as a response to maintain the acid-base balance after exceeding RCP leads to cerebral vasoconstriction and therefore also in cerebral oxygenation. These findings reinforce and expand the knowledge on cerebral oxygenation during whole-body endurance exercise. Future research should examine whether maintaining/improving PFC oxygenation can improve endurance performance.

## Data Availability Statement

The original contributions presented in the study are included in the article/supplementary material, further inquiries can be directed to the corresponding author/s.

## Author Contributions

JDW performed the design of the search strategy. Subsequently, MP, JVC, JH, JD-G, and BR revised the design. JDW and JH did screening on title and abstract, while JDW, MP, JH, JVC, and BR conducted full text screening. JDW, MP, and JH first conducted the data analysis. JVC and BR later revised and updated the data analysis. JDW and JH performed quality assessment and also designed the quality assessment table together with MP. MV and JDW performed calculations for exercise intensity and performance level. JDW wrote the first draft of the manuscript, which was later altered by MP, JH, MV, PH, JD-G, JVC, BR, and RM. All authors read, revised, and approved the final manuscript.

## Conflict of Interest

The authors declare that the research was conducted in the absence of any commercial or financial relationships that could be construed as a potential conflict of interest.

## Publisher’s Note

All claims expressed in this article are solely those of the authors and do not necessarily represent those of their affiliated organizations, or those of the publisher, the editors and the reviewers. Any product that may be evaluated in this article, or claim that may be made by its manufacturer, is not guaranteed or endorsed by the publisher.
